# The Association of Serum Anti-Lysosomal-Associated Membrane Protein-2 Antibody with Vasculitis Combined with Hypertension

**DOI:** 10.1155/2022/9656560

**Published:** 2022-03-18

**Authors:** Bin Zhu, Xintian Cai, Qing Zhu, Ting Wu, Shasha Liu, Shanshan Liu, Jing Hong, Nanfang Li

**Affiliations:** ^1^Hypertension Center of People's Hospital of Xinjiang Uygur Autonomous Region, Xinjiang Hypertension Institute, National Health Committee Key Laboratory of Hypertension Clinical Research, Key Laboratory of Xinjiang Uygur Autonomous Region, Xinjiang Clinical Medical Research Center for Hypertension Diseases, Urumqi, China; ^2^Department of Cardiology, Xijing Hospital, Air Force Medical University, Xi'an, China; ^3^Xinjiang Medical University, Urumqi, China

## Abstract

The aim of this study was to explore the association of serum anti-lysosomal-associated membrane protein-2 (anti-LAMP-2) antibody with vasculitis combined with hypertension (VAS-HTN). A total of 51 VAS-HTN patients, 46 essential hypertension (EH) patients, and 46 healthy controls (HC) were included in the study. Serum anti-LAMP-2 antibody levels are increased in VAS-HTN patients as compared with EH and HC (all *P* < 0.05). Serum anti-LAMP-2 antibody levels were significantly higher in active stage patients than those in non-active stage patients and HC (all *P* < 0.05). The correlation analysis showed a significant positive correlation between serum anti-LAMP-2 antibody levels and the Birmingham Vasculitis Activity Score (BVAS) and hypersensitive C-reactive protein (Hs-CRP) (all *P* < 0.05). Among the subsets of VAS-HTN, the levels of serum anti-LAMP-2 antibody were remarkably higher in all VAS-HTN subsets compared with HC (all *P* < 0.05). More interestingly, the levels of serum anti-LAMP-2 antibody were remarkably increased in polyarteritis nodosa (PAN) patients compared with ANCA-associated vasculitis and Takayasu arteritis patients (all *P* < 0.05). In addition, there was a significant positive correlation between serum anti-LAMP-2 antibody levels and BAVS and Hs-CRP in PAN patients (all *P* < 0.05). Multivariate logistic regression analysis showed that the anti-LAMP-2 antibody was independently associated with VAS-HTN. The levels of serum anti-LAMP-2 antibody were remarkably increased in VAS-HTN patients compared to EH and HC and might reflect the disease activity. The anti-LAMP-2 antibody may be a potential biomarker for diagnosis and estimating the disease activity in VAS-HTN.

## 1. Introduction

Hypertension is a global public health problem. It is currently the main contributor to the global burden of disease [1]. Numerous studies have confirmed that an increasing number of patients with hypertension is attributed to various diseases (e.g., kidney disease, primary aldosteronism, and vasculitis) [2–5]. Among them, the incidence of vasculitis is very insidious and harmful [6]. Because of the particularity of etiology, conventional antihypertensive therapy is not feasible. Furthermore, the target organ (renal, lung, heart, brain, etc.) damage caused by vasculitis is very similar and more serious to that caused by hypertension [7, 8]. Consequently, differential diagnosis of patients with vasculitis combined with hypertension (VAS-HTN) is extremely important in the hypertensive population.

Vasculitis is a disabling disease featuring inflammatory injury and destruction that affects all sizes of vessels in the body [9]. Recently, the incidence of vasculitis has gradually increased, which has brought a heavy economic burden to society [10, 11]. Hypertension is a common clinical feature of vasculitis [12, 13]. In clinical, hypertension specialists will also encounter patients with vasculitis. However, vasculitis presents with a wide range of clinical presentations and lacks specific laboratory indicators. The identification and evaluation of disease in vasculitis is a tremendous challenge. Therefore, finding novel biomarkers is very important for VAS-HTN patients to be identified from hypertensive populations and to create conditions for future treatment.

Lysosomal-associated membrane protein-2 (LAMP-2) is a transmembrane protein that participates in molecular chaperone-mediated autophagy and the presentation of intracellular antigens [14–17]. Kain et al.'s study found that LAMP-2 was expressed on the surface of neutrophils and was considered the anti-neutrophil cytoplasmic antibody (ANCA) antigen [18]. Recent studies suggest that anti-LAMP-2 antibody has been involved in the pathogenesis of vasculitis [19–21]. Some research showed that the levels of serum anti-LAMP-2 antibody were observed to increase in small vessel vasculitis [22–24]. Nevertheless, other research demonstrated that the levels of serum anti-LAMP-2 antibody were similar in ANCA-associated vasculitis (AAV) patients compared to healthy controls (HC) [22, 23, 25]. Therefore, whether the anti-LAMP-2 antibody is associated with vasculitis remains controversial. Furthermore, serum anti-LAMP-2 antibody levels have not been evaluated in VAS-HTN, particularly in Takayasu arteritis (TA) and polyarteritis nodosa (PAN). The goal of this study was to examine whether there is a relationship between serum anti-LAMP-2 antibody levels and VAS-HTN.

## 2. Methods

### 2.1. Study Subjects

**Figure 1:**
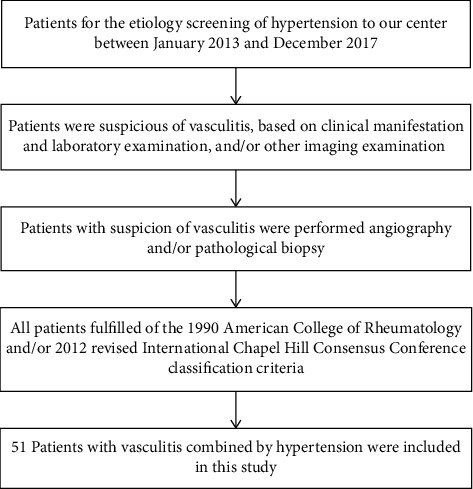
The screening and diagnostic flow chart for vasculitis combined with hypertension.

**Figure 2:**
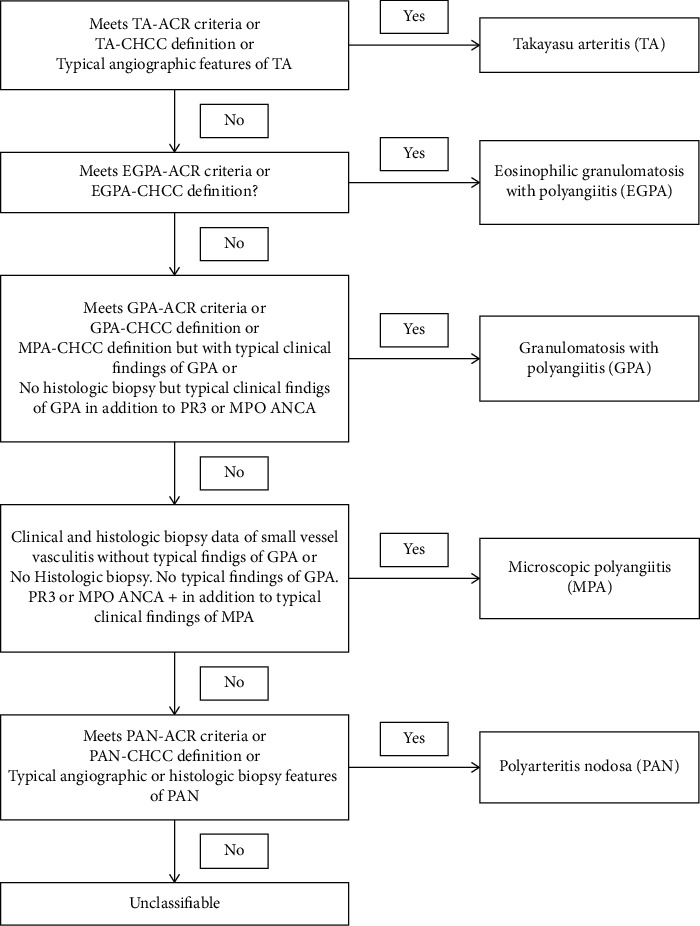
The criteria for the diagnosis of vasculitis.

All enrolled VAS-HTN patients were seen at the People's Hospital of Xinjiang Uygur Autonomous Region from January 2013 to December 2017, and the diagnosis was made based on clinical presentation, laboratory tests, imaging examinations, or/and pathological biopsies. Finally, 51 VAS-HTN patients (including 24 PAN, 20 AAV, and 7TA) were included in this study. The diagnostic flow chart of VAS-HTN screening is shown in Figure 1. All vasculitis patients fulfilled the 1990 ACR and/or 2012 revised CHCC classification criteria [26–30]. The criteria for the diagnosis of vasculitis are shown in Figure 2. Also, 46 patients with age- and sex-matched essential hypertension (EH) and 46 HC subjects were include in the study.

### 2.2. Data Collection and Measurements

All clinical information data is derived from the electronic medical records of patients during their hospitalization. Details of the data collection and measurements can be found in previously published papers [31, 32] and online supplemental material.

### 2.3. Definitions of Disease Activity

Disease activity was assessed according to the third version of the Birmingham Vasculitis Activity Score (BVAS) [33]. The BVAS = 0 was considered inactive in VAS-HTN patients, and the BVAS ≥ 1 was considered active in VAS-HTN patients.

### 2.4. Definitions of Renal Injury

Renal injury was defined as any proteinuria and/or hematuria and/or elevated serum Scr. Proteinuria was defined as a urine routine of more than 1+ and/or urine containing more than 0.15 g of protein was collected for 24 hours. Hematuria was defined as more than 5 red blood cells per high magnification view in the urine sediment. Elevated serum Scr was defined as a female Scr > 84 *μ*mol/L or a male Scr > 104 *μ*mol/L [31].

### 2.5. Serum Sample Collection and Anti-LAMP-2 Antibody Measurement

Blood samples from all participants were drawn into procoagulation tubes, and the serum was collected after centrifugation at 3000 g at 4°C for 15 minutes as soon as possible. The serum was stored in a refrigerator at −80°C for standby. The levels of serum anti-LAMP-2 antibodies were measured using a commercial ELISA kit according to the manufacturer's instructions (Uscn Life Sciences, Wuhan, China).

### 2.6. Statistical Analysis

SPSS software version 20.0 and GraphPad Prism software 5.0 were employed for statistical analysis and graphing. Mean ± standard deviation, or median and interquartile range, was used to present continuous variables, as appropriate. Categorical variables are expressed as totals and percentages. The *t*-test/one-way ANOVA or rank-sum test is used for comparisons across groups, as appropriate. Spearman or Pearson rank correlations were used to assess correlations. Binary logistic regression was used to determine independent factors for the diagnosis of VAS-HTN. Receiver operating characteristic (ROC) curve analysis was used to determine the optimal cutoff value for anti-LAMP-2 antibodies. *P* value <0.05 was regarded as significant.

## 3. Results

### 3.1. Clinical and Laboratory Features

The mean age of the patients with VAS-HTN in this study was 40.22 ± 9.62 years, and women accounted for 43.1% of all patients. Among the VAS-HTN patients, 24 were diagnosed with PAN, 20 with AAV, and 7 with TA. 16 patients were in the inactive phase and 35 patients were in the active phase according to BVAS assessment. The basic characteristics of 51 VAS-HTN patients, 46 EH patients, and 46 HC patients are presented in Tables 1 and 2.

### 3.2. Serum Anti-LAMP-2 Antibody Levels

**Figure 3:**
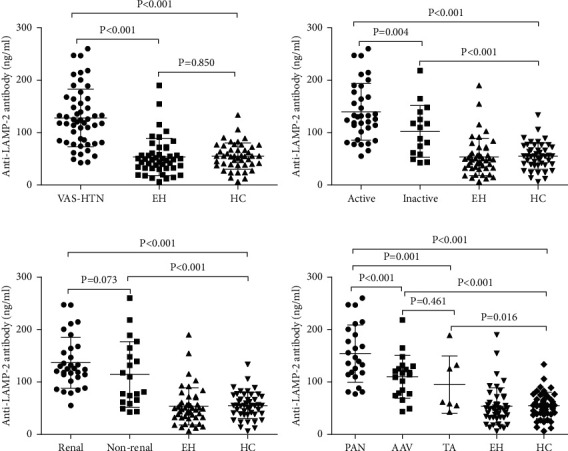
The levels of serum anti-LAMP-2 antibody in different groups. (a) In VAS-HTN patients and HC. (b) In VAS-HTN patients with the active stage and inactive stage. (c) In VAS-HTN patients with renal injury and non-renal injury. (d) In VAS-HTN subsets.

The levels of anti-LAMP-2 antibody are increased in VAS-HTN patients as compared with EH and HC (VAS-HTN vs. EH: (127.98 ± 54.98) vs. (53.59 ± 35.51) ng/ml, *P* < 0.001; VAS-HTN vs. HC: (127.98 ± 54.98) vs. (55.21 ± 25.09) ng/ml, *P* < 0.001) (Figure 3(a)). Also, no significant differences were found in serum anti-LAMP-2 antibody levels between EH and HC ((53.59 ± 35.51) vs. (55.21 ± 25.09) ng/ml, *P*=0.850) (Figure 3(a)).

Serum anti-LAMP-2 antibody levels were remarkably higher in VAS-HTN patients with active stage and inactive stage than in HC (active vs. HC: (139.65 ± 54.02) vs. (55.21 ± 25.09) ng/ml, *P* < 0.001; inactive vs. HC: (102.47 ± 49.48) vs. (55.21 ± 25.09) ng/ml, *P* < 0.001) (Figure 3(b)). Furthermore, we found that serum anti-LAMP-2 antibody levels were remarkably increased in VAS-HTN patients with active stages compared with those in inactive stage patients ((139.65 ± 54.02) vs. (102.47 ± 49.48) ng/ml, *P*=0.004) (Figure 3(b)).

Compared with HC, renal injury and non-renal injury patients had significantly increased the levels of anti-LAMP-2 antibody (renal vs. HC (136.75 ± 48.53) ng/ml vs. (55.21 ± 25.09), *P* < 0.001; non-renal vs. HC (114.39 ± 62.56) ng/ml vs. (55.21 ± 25.09) ng/ml, *P* < 0.001) (Figure 3(c)). Serum anti-LAMP-2 antibody levels were slightly but not significantly higher in VAS-HTN patients with renal injury than in those with non-renal injury ((136.75 ± 48.53) ng/ml vs. (114.39 ± 62.56) ng/ml, *P*=0.073) (Figure 3(c)).

Among the subsets of VAS-HTN, the levels of serum anti-LAMP-2 antibody were remarkably increased in all VAS-HTN subsets compared with HC (AAV vs. HC: (107.90 ± 41.13) vs. (55.21 ± 25.09) ng/ml, *P* < 0.001; PAN vs. HC: (154.34 ± 54.65) vs. (55.21 ± 25.09) ng/ml, *P* < 0.001; TA vs. HC: (94.98 ± 54.65) vs. (55.21 ± 25.09) ng/ml, *P*=0.016) (Figure 3(d)). More interestingly, serum anti-LAMP-2 antibody levels were significantly increased in PAN patients compared with AAV and TA patients (PAN vs. AAV: (154.34 ± 54.65) vs. (107.90 ± 41.13) ng/ml, *P* < 0.001; PAN vs. TA: (154.34 ± 54.65) vs. (94.98 ± 54.65) ng/ml, *P*=0.001) (Figure 3(d)). There was no difference found in serum anti-LAMP-2 antibody levels between AAV and TA patients ((107.90 ± 41.13) vs. (94.98 ± 54.65) ng/ml, *P*=0.461) (Figure 3(d)).

### 3.3. Correlations between Serum Anti-LAMP-2 Antibody Levels and Clinical and Laboratory Parameters

**Figure 4:**
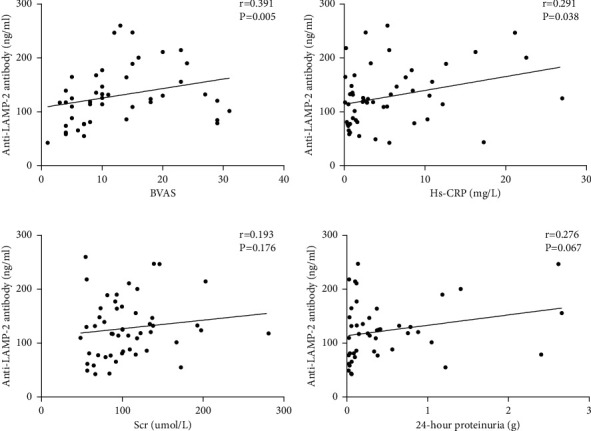
Correlations of anti-LAMP-2 antibody levels with BVAS, Hs-CRP, Scr, and 24-hour proteinuria in patients with vasculitis combined with hypertension.

**Figure 5:**
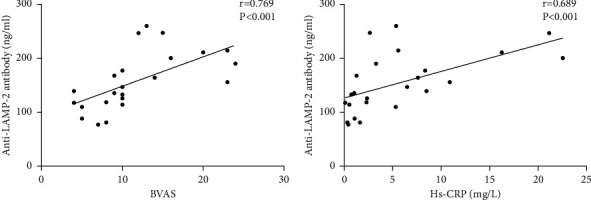
Correlations of anti-LAMP-2 antibody levels with BVAS and Hs-CRP in patients with polyarteritis nodosa.

We evaluated whether serum anti-LAMP-2 antibody levels are in correlation with clinical and laboratory parameters in VAS-HTN patients.. The correlation analysis showed that there was a positive correlation between serum anti-LAMP-2 antibody levels and BVAS (*r* = 0.391, *P*=0.005), Hs-CRP (*r* = 0.291, *P*=0.038) (Figures 4(a) and 4(b)). However, there was no correlation between serum anti-LAMP-2 antibody levels and Scr (*r* = 0.193, *P*=0.176), 24-hour proteinuria (*r* = 0.276, *P*=0.067) (Figures 4(c) and 4(d)). In addition, we further analyzed the relationship between serum anti-LAMP-2 antibody levels and clinical and laboratory parameters in PAN patients. The results suggest that there was a significant positive correlation between serum anti-LAMP-2 antibody levels and BAVS, Hs-CRP (Figures 5(a) and 5(b)).

As shown in Table 3, multivariate logistic regression analysis showed that anti-LAMP-2 antibody (OR: 1.028, *P*=0.004) and Scr (OR: 1.079, *P*=0.003) were all independently associated with VAS-HTN.

### 3.4. ROC Curve Analysis

The diagnostic performance of serum anti-LAMP-2 antibody levels for detecting VAS-HTN patients using the ROC curves. The best cutoff values for serum anti-LAMP-2 antibody levels were determined using the highest value of the Youden index. The results showed that the best cutoff value, sensitivity, and specificity were at 76.99 ng/ml, 84.3%, and 83.7%. When assessing the active stage with serum anti-LAMP-2 antibody levels in VAS-HTN patients, a cutoff value of 117.58 ng/ml with a sensitivity of 65.7% and a specificity of 68.8% was observed according to ROC curve analysis. Further evaluation of patients with the VAS-HTN subgroup revealed that the optimal anti-LAMP-2 antibody cutoff value for identifying PAN and VAS-HTN was 132.38 ng/ml, with a sensitivity of 62.5% and specificity of 85.2%. All estimated values are shown in Table 4.

## 4. Discussion

Recently, it has been reported that anti-LAMP-2 antibody was associated with small-vessel vasculitis. Kain et al. research findings suggest that LAMP-2 autoantibodies can be detected in most AAV patients, and possibly related to the disease activity [34]. However, Roth et al. concluded that LAMP-2 antibodies may be present at very low titers in a minority of AAV patients [25]. The reason for the difference between the two results may be due to the different case selection and detection methods [35]. Therefore, the role of anti-LAMP-2 antibodies in vasculitis is still far from clear. As far as we know, no published research has reported the relationship between serum anti-LAMP-2 antibody levels and VAS-HTN in hypertensive populations.

In this study, all the selected VAS-HTN patients complained of hypertension and visited our center. Vasculitis included seven categories according to the 2012 CHCC classification criteria [26]. Our study has three types of vasculitis, which were included in this study. This also shows that these three types of vasculitis are more likely to lead to hypertension. This is consistent with some reports [12, 13]. We investigated further the serum anti-LAMP-2 antibody levels in VAS-HTN patients, EH, and HC based on ELISA. Our results showed that the levels of serum anti-LAMP-2 antibody were significantly higher in VAS-HTN patients compared with EH and HC. Furthermore, we found that the active stage of VAS-HTN patients was closely related to the elevated anti-LAMP-2 antibody levels. Multivariate logistic regression analysis showed that the anti-LAMP-2 antibody was an independent factor associated with VAS-HTN. On account of these results, we believe that the anti-LAMP-2 antibody could be an independent factor of VAS-HTN and might reflect the disease activity. In addition, the levels of serum anti-LAMP-2 antibody in patients with the inactive stage still remained elevated compared with HC. These outcomes could possibly be a reflection of the persistent low-grade inflammatory activity or vascular wall lesions in spite of relatively normal clinical and laboratory parameters.

Peschel et al. discovered autoantibodies to hLAMP-2 that conjugate to natural glomeruli, suggesting an important role in the pathogenesis of ANCA-negative oligoimmune focal necrotizing glomerulonephritic lesions [36]. Therefore, we investigated whether serum anti-LAMP-2 antibody related to renal injury in VAS-HTN. However, whether it is directly engaged in the renal damage process is unclear. As a next step, we need to expand the volume of samples to further prove this result.

In the present study, we found that all VAS-HTN subsets presented increased markedly serum anti-LAMP-2 antibody levels than in EH and HC. To our surprise, the levels of serum anti-LAMP-2 antibody were increased significantly in PAN patients compared with AAV and TA patients. Moreover, our results showed that serum anti-LAMP-2 antibody levels were significantly positively correlated with BAVS and Hs-CRP. Our results indicate that anti-LAMP-2 antibody could play a role in the pathogenesis of PAN and can reflect the disease activity. Further studies examining anti-LAMP-2 antibody levels during the course of disease in PAN are needed.

The quality of the anti-LAMP-2 antibody as a biomarker was evaluated on account of ROC analysis. For the diagnosis of VAS-HTN, the optimal cutoff values of serum anti-LAMP-2 antibody were determined to be 76.99 ng/ml. We further assess the active stage with serum anti-LAMP-2 antibody levels in VAS-HTN patients, with a cutoff value of 117.58 ng/ml with a sensitivity of 65.7% and a specificity of 68.8%. Furthermore, the analysis of patients with VAS-HTN subsets revealed that the best anti-LAMP-2 antibody cutoff value for differentiating PAN from VAS-HTN is 132.38  ng/ml with 62.5% sensitivity and 85.2% specificity. It suggests that anti-LAMP-2 antibody might serve as a potential biomarker for diagnosing VAS-HTN and to assist in the assessment of the disease activity. In addition, we believe that the detection of serum anti-LAMP-2 antibody may be a feasible method to distinguish PAN from AAV and TA.

In the present study, our data seem robust enough to conclude that the anti-LAMP-2 antibody is a suitable biomarker for the clinical diagnosis and disease assessment for VAS-HTN. It is helpful for the differential diagnosis of SV in the hypertensive population, the rational formulation of the treatment plan, the effective evaluation of treatment effect, the prediction of recurrence, and the better clinical management of VAS-HTN patients in the future. However, our study has some limitations that need to be considered. First, the present study failed to investigate the dynamics of serum anti-LAMP-2 antibody levels. Further longitudinal studies are needed to observe the dynamic changes. Second, the low number of patients requires further expanding the sample size. Finally, our study did not evaluate the comparison of diagnostic performance between anti-LAMP-2 and other vasculitis diagnosis markers, such as anti-endothelial cell antibody (AECA). Further studies are needed to verify the diagnostic performance of anti-LAMP-2 antibodies in the future.

## 5. Conclusion

The study suggests that anti-LAMP-2 antibodies were higher in patients with VAS-HTN compared to EH and HC, and might reflect the disease activity, particularly in patients with PAN. The anti-LAMP-2 antibody may be a potential biomarker for diagnosis and estimating the disease activity in VAS-HTN.

## Figures and Tables

**Table 1:** Demographic and laboratory features.

Variables	VAS-HTN (*n* = 51)	EH (*n* = 46)	HC (*n* = 46)	*P* value	AAV (*n* = 20)	PAN (*n* = 24)	TA (*n* = 7)
Demographic features							
Age (years)	40.22 ± 9.62	43.74 ± 9.22	42.61 ± 15.87	0.287	42.50 ± 8.78	38.54 ± 9.45	38.00 ± 12.29
Females, *n* (%)	22 (43.1)	15 (32.6)	26 (56.5)	0.121	11 (55.0)	7 (29.2)	4 (57.1)
SBP (mmHg)	164.65 ± 27.33	142.50 ± 17.59	118.26 ± 11.34	<0.001	161.95 ± 29.09	167.08 ± 26.16	164.00 ± 29.54
DBP (mmHg)	104.20 ± 19.26	88.11 ± 11.31	73.13 ± 8.55	<0.001	99.05 ± 24.10	110.00 ± 14.59	99.00 ± 13.40
BMI (kg/m^2^)	25.76 ± 3.99	27.05 ± 4.25	23.32 ± 3.03	<0.001	25.46 ± 3.48	26.39 ± 4.58	24.44 ± 3.14
Laboratory features							
ESR (mm/h)	18.43 ± 14.21	10.22 ± 7.77	9.23 ± 5.15	0.003	20.80 ± 15.43	15.25 ± 15.95	22.57 ± 14.41
Scr (umol/L)	106.62 ± 45.43	66.33 ± 16.56	72.36 ± 12.44	<0.001	107.33 ± 55.64	108.79 ± 38.51	97.14 ± 43.30
WBC (×10^9^/L)	7.37 ± 2.83	6.22 ± 1.43	6.53 ± 1.83	0.026	6.70 ± 2.79	7.33 ± 1.80	9.42 ± 4.85
HB (g/L)	138.59 ± 20.95	145.17 ± 11.99	150.38 ± 15.18	0.015	134.35 ± 25.09	145.58 ± 15.96	126.71 ± 16.31
PLT (×109/L)	245.10 ± 106.73	254.65 ± 67.74	262.50 ± 44.85	0.552	252.40 ± 95.19	222.63 ± 80.71	301.29 ± 188.09
ANCA (+), *n* (%)	8 (15.7)	0 (0)	0 (0)	<0.001	7 (35.0)	0 (0)	1 (14.3)
Hs-CRP (mg/L)	2.82 (0.91 to 8.35)	1.04 (0.42 to 2.64)	—	0.001	2.64 (0.77 to 7.72)	2.97 (1.04 to 8.17)	5.59 (0.72 to 26.99)
Proteinuria (+), *n* (%)	20 (39.2)	0 (0)	0 (0)	<0.001	7 (35.0)	11 (45.8)	2 (28.6)
Hematuria (+), *n* (%)	7 (13.7)	0 (0)	0 (0)	<0.001	3 (15.0)	3 (12.5)	1 (14.3)
24-hour proteinuria (g)	0.20 (0.06 to 0.61)	0.06 (0.04 to 0.09)	—	<0.001	0.12 (0.05 to 0.37)	0.28 (0.12 to 0.71)	0.23 (0.03 to 0.79)
Anti-LAMP-2 (ng/ml)	127.98 ± 54.98	53.59 ± 35.51	55.21 ± 25.09	<0.001	107.90 ± 41.13	154.34 ± 54.65	94.98 ± 54.65

Continuous variables are shown as median and interquartile range or as mean ± standard deviation.

**Table 2:** Clinical features of patients with vasculitis combined with hypertension.

Clinical features	VAS-HTN (*n* = 51)	AAV (*n* = 20)	PAN (*n* = 24)	TA (*n* = 7)
Headache, *n* (%)	30 (58.8)	9 (45.0)	18 (75.0)	3 (42.9)
Constitutional symptoms, *n* (%)	32 (62.7)	13 (65.0)	15 (62.5)	4 (57.1)
Nervous systems, *n* (%)	4 (7.8)	2 (10.0)	1 (4.2)	1 (14.3)
Renal systems, *n* (%)	31 (60.8)	12 (60.0)	16 (66.7)	3 (42.9)
Cutaneous, *n* (%)	4 (7.8)	0 (0)	3 (12.5)	1 (14.3)
Arthritis/joint pain, *n* (%)	2 (3.9)	0 (0)	2 (8.3)	0 (0)
Eye, *n* (%)	15 (29.4)	7 (35.0)	7 (29.2)	1 (14.3)
Ear nose throat, *n* (%)	0 (0)	0 (0)	0 (0)	0 (0)
Pulmonary system, *n* (%)	9 (17.6)	9 (45.0)	0 (0)	0 (0)
Alimentary system, *n* (%)	6 (11.8)	3 (15.0)	2 (8.3)	1 (14.3)
Cardiovascular system, *n* (%)	10 (19.6)	5 (25.0)	5 (20.8)	0 (0)
Active disease, *n* (%)	35 (68.6)	13 (65.0)	19 (79.2)	3 (42.9)
BVAS	11.35 ± 8.61	12.35 ± 10.82	11.21 ± 6.36	9.00 ± 9.07

Continuous variables are shown as mean ± standard deviation.

**Table 3:** Logistic regression analysis of vasculitis combined with hypertension patients versus controls.

Variable	Univariate			Multivariate		
	OR	95% CI	*P* value	OR	95% CI	*P* value
Age	0.978	0.949–1.007	0.133	—	—	—
Sex	1.06	0.532–2.113	0.869	—	—	—
SBP	1.07	1.046–1.096	<0.001	1.046	0.992–1.103	0.095
DBP	1.1	1.064–1.138	<0.001	1.014	0.943–1.090	0.704
BMI	1.035	0.952–1.126	0.421	—	—	—
WBC	1.245	1.044–1.485	0.015	0.863	0.579–1.288	0.471
HB	0.97	0.48–0.993	0.012	0.958	0.895–1.026	0.221
PLT	0.998	0.993–1.002	0.368	—	—	—
Hs-CRP	1.22	1.055–1.411	0.007	1.029	0.819–1.293	0.804
ESR	1.082	1.036–1.129	<0.001	1.017	0.914–1.132	0.759
Scr	1.063	1.038–1.088	<0.001	1.079	1.026–1.135	0.003
Anti-LAMP-2	1.044	1.029–1.059	<0.001	1.028	1.009–1.047	0.004

OR: odds ratio; CI, confidence interval.

**Table 4:** Estimated value of serum anti-LAMP-2 antibody levels based on the cohort.

Estimated value	VAS-HTN	Active	Subsets (PAN)
Sensitivity (%)	84.3 (71.4 to 93.0)	65.7 (47.8 to 80.9)	62.5 (40.6 to 81.2)
Specificity (%)	83.7 (74.5 to 90.6)	68.8 (41.3 to 89.0)	85.2 (66.3 to 95.7)
PPV (%)	74.1 (61.0 to 84.7)	82.1 (63.1 to 93.9)	78.9 (54.4 to 93.9)
NPV (%)	90.6 (82.3 to 95.8)	47.8 (26.8 to 69.4)	71.9 (53.3 to 86.3)
PLR	5.17 (3.2 to 8.3)	2.10 (1.0 to 4.5)	4.22 (1.6 to 11.0)
NLR	0.19 (0.1 to 0.4)	0.50 (0.3 to 0.9)	0.44 (0.3 to 0.8)
AUC	0.90 (0.84 to 0.94)	0.70 (0.55 to 0.82)	0.77 (0.63 to 0.87)
YI	0.680	0.345	0.477

Values in parentheses are 95% CI.

## Data Availability

Data supporting the results of this research are accessible from the first author.

## References

[1] Wang Z., Chen Z., Zhang L. (2018). Status of hypertension in China: results from the China hypertension survey, 2012–2015. *Circulation*.

[2] Pugh D., Gallacher P. J., Dhaun N. (2019). Management of hypertension in chronic kidney disease. *Drugs*.

[3] Tokunou T., Ando S.-I. (2020). Recent advances in the management of secondary hypertension-obstructive sleep apnea. *Hypertension Research*.

[4] Zennaro M.-C., Boulkroun S., Fernandes-Rosa F. L. (2020). Pathogenesis and treatment of primary aldosteronism. *Nature Reviews Endocrinology*.

[5] Eicken S., Gugger M., Marti P. (2012). Glomerulonephritis und Vaskulitis als Ursacheneiner arteriellen Hypertonie. *Therapeutische Umschau*.

[6] Scott D. G. I., Watts R. A. (2013). Epidemiology and clinical features of systemic vasculitis. *Clinical and Experimental Nephrology*.

[7] Blaustein D., Kumbar L., Srivastava M., Avram M. M. (2004). Polyarteritis nodosa presenting as isolated malignant hypertension. *American Journal of Hypertension*.

[8] Horino T., Ichii O., Matsumoto T., Terada Y. (2020). Takayasu arteritis presenting as renovascular hypertension. *Journal of Clinical Rheumatology*.

[9] Ferro F., Quartuccio L., Monti S. (2021). One year in review 2021: systemic vasculitis. *Clinical & Experimental Rheumatology*.

[10] Knight A., Ekbom A., Brandt L., Askling J. (2006). Increasing incidence of Wegener’s granulomatosis in Sweden, 1975-2001. *Journal of Rheumatology*.

[11] Trieste L., Palla I., Baldini C. (2012). Systemic vasculitis: how little we know about their societal and economic burden. *Clinical & Experimental Rheumatology*.

[12] Qi Y., Yang L., Zhang H. (2018). The presentation and management of hypertension in a large cohort of Takayasu arteritis. *Clinical Rheumatology*.

[13] Cohen L., Guillevin L., Meyrier A., Bironne P., Blétry O., Godeau P. (1986). Malignant hypertension and polyarteritis nodosa: clinical and biological data in 165 patients. *Archives des Maladies du Coeur et des Vaisseaux*.

[14] Eskelinen E.-L., Illert A. L., Tanaka Y. (2002). Role of LAMP-2 in lysosome biogenesis and autophagy. *Molecular Biology of the Cell*.

[15] Huynh K. K., Eskelinen E.-L., Scott C. C., Malevanets A., Saftig P., Grinstein S. (2007). LAMP proteins are required for fusion of lysosomes with phagosomes. *The EMBO Journal*.

[16] Saftig P., Beertsen W., Eskelinen E.-L. (2008). LAMP-2: a control step for phagosome and autophagosome maturation. *Autophagy*.

[17] Schmid D., Münz C. (2007). Innate and adaptive immunity through autophagy. *Immunity*.

[18] Kain R., Matsui K., Exner M. (1995). A novel class of autoantigens of anti-neutrophil cytoplasmic antibodies in necrotizing and crescentic glomerulonephritis: the lysosomal membrane glycoprotein h-lamp-2 in neutrophil granulocytes and a related membrane protein in glomerular endothelial cells. *Journal of Experimental Medicine*.

[19] Takeuchi S., Kimura S., Soma Y. (2013). Lysosomal-associated membrane protein-2 plays an important role in the pathogenesis of primary cutaneous vasculitis. *Rheumatology*.

[20] Willcocks L. C., Lyons P. A., Rees A. J., Smith K. G. (2010). The contribution of genetic variation and infection to the pathogenesis of ANCA-associated systemic vasculitis. *Arthritis Research and Therapy*.

[21] Tang S., Zhang Y., Yin S.-W. (2015). Neutrophil extracellular trap formation is associated with autophagy-related signalling in ANCA-associated vasculitis. *Clinical and Experimental Immunology*.

[22] Kawakami T., Takeuchi S., Arimura Y., Soma Y. (2012). Elevated antilysosomal-associated membrane protein-2 antibody levels in patients with adult Henoch-Schönlein purpura. *British Journal of Dermatology*.

[23] Kawakami T., Ishizu A., Arimura Y., Soma Y. (2013). Serum anti-lysosomal-associated membrane protein-2 antibody levels in cutaneous polyarteritis nodosa. *Acta Dermato-Venereologica*.

[24] Kain R., Exner M., Brandes R. (2008). Molecular mimicry in pauci-immune focal necrotizing glomerulonephritis. *Nature Medicine*.

[25] Roth A. J., Brown M. C., Smith R. N. (2012). Anti-LAMP-2 antibodies are not prevalent in patients with antineutrophil cytoplasmic autoantibody glomerulonephritis. *Journal of the American Society of Nephrology*.

[26] Jennette J. C., Falk R. J., Bacon P. A. (2012). 2012 revised international chapel hill consensus conference nomenclature of vasculitides. *Arthritis & Rheumatism*.

[27] Leavitt R. Y., Fauci A. S., Bloch D. A. (1990). The American College of Rheumatology 1990 criteria for the classification of Wegener’s granulomatosis. *Arthritis & Rheumatism*.

[28] Masi A. T., Hunder G. G., Lie J. T. (1990). The American College of Rheumatology 1990 criteria for the classification of Churg-Strauss syndrome (allergic granulomatosis and angiitis). *Arthritis & Rheumatism*.

[29] Lightfoot R. W., Michel B. A., Bloch D. A. (1990). The American College of Rheumatology 1990 criteria for the classification of polyarteritis nodosa. *Arthritis & Rheumatism*.

[30] Arend W. P., Michel B. A., Bloch D. A. (1990). The American College of Rheumatology 1990 criteria for the classification of Takayasu arteritis. *Arthritis & Rheumatism*.

[31] Zhu B., Li N., Zhu Q. (2019). Association of serum high mobility group box 1 levels with disease activity and renal involvement in patients with systemic vasculitis. *Medicine*.

[32] Li N., Zhu B., Zhu Q. (2019). Serum lysosomal-associated membrane protein-2 levels are increased in small and medium-vessel vasculitis, especially in polyarteritis nodosa. *Clinical & Experimental Rheumatology*.

[33] Mukhtyar C., Lee R., Brown D. (2009). Modification and validation of the Birmingham vasculitis activity Score (version 3). *Annals of the Rheumatic Diseases*.

[34] Kain R., Tadema H., McKinney E. F. (2012). High prevalence of autoantibodies to hLAMP-2 in anti-neutrophil cytoplasmic antibody-associated vasculitis. *Journal of the American Society of Nephrology*.

[35] Flint S. M., Savage C. O. S. (2012). Anti-LAMP-2 autoantibodies in ANCA-associated pauci-immune glomerulonephritis. *Journal of the American Society of Nephrology*.

[36] Peschel A., Basu N., Benharkou A. (2014). Autoantibodies to hLAMP-2 in ANCA-negative pauci-immune focal necrotizing GN. *Journal of the American Society of Nephrology*.

